# Kyasanur Forest disease virus infection activates human vascular endothelial cells and monocyte-derived dendritic cells

**DOI:** 10.1038/s41426-018-0177-z

**Published:** 2018-11-07

**Authors:** Jana Sirmarova, Jiri Salat, Martin Palus, Vaclav Hönig, Helena Langhansova, Michael R. Holbrook, Daniel Ruzek

**Affiliations:** 1 Department of Virology, Veterinary Research Institute, Hudcova 70, CZ-62100, Brno, Czech Republic; 2 Institute of Parasitology, Biology Centre of the Czech Academy of Sciences, Branisovska 31, CZ-37005 Ceske Budejovice, Czech Republic; 3 Faculty of Science, University of South Bohemia, Branisovska 31, CZ-37005 Ceske Budejovice, Czech Republic; 4 NIAID Integrated Research Facility, 8200 Research Plaza, Ft. Detrick, Frederick, MD 21702 USA

## Abstract

Kyasanur Forest disease virus (KFDV) is a highly pathogenic tick-borne flavivirus enzootic to India. In humans, KFDV causes a severe febrile disease. In some infected individuals, hemorrhagic manifestations, such as bleeding from the nose and gums and gastrointestinal bleeding with hematemesis and/or blood in the stool, have been reported. However, the mechanisms underlying these hemorrhagic complications remain unknown, and there is no information about the specific target cells for KFDV. We investigated the interaction of KFDV with vascular endothelial cells (ECs) and monocyte-derived dendritic cells (moDCs), which are key targets for several other hemorrhagic viruses. Here, we report that ECs are permissive to KFDV infection, which leads to their activation, as demonstrated by the upregulation of E-selectin, intercellular adhesion molecule 1, and vascular cell adhesion molecule 1 at the mRNA and protein levels. Increased expression of these adhesive molecules correlated with increased leukocyte adhesion. Infected ECs upregulated the expression of interleukin (IL)-6 but not IL-8. Additionally, moDCs were permissive to KFDV infection, leading to increased release of IL-6 and tumor necrosis factor-α. Supernatants from KFDV-infected moDCs caused EC activation, as measured by leukocyte adhesion. The results indicate that ECs and moDCs can be targets for KFDV and that both direct and indirect mechanisms can contribute to EC activation.

## Introduction

Kyasanur Forest disease (KFD) virus (KFDV) is an emerging tick-borne pathogen and a member of the genus *Flavivirus* within the family *Flaviviridae*. KFDV is prevalent in southern parts of India, where it causes acute hemorrhagic illness in humans and wild nonhuman primates^1,2^. Transmission of KFDV to humans occurs after an infected tick bite (mainly *Haemaphysalis spinigera*) or contact with an infected animal, mostly sick or dead monkeys. Annual numbers of human cases of KFD range from 400 to 500 in endemic regions, with a case fatality rate of 3–5% (up to 10% during outbreaks); however, it is believed that these numbers are largely underestimated^2^. Diseases similar to KFD have been reported in Saudi Arabia and China, and recent outbreaks of KFD also have been documented in other parts of India, indicating that the infection is expanding to new areas^3,4^.

Clinically, KFD resembles Omsk hemorrhagic fever, another tick-borne hemorrhagic disease^5,6^. The incubation period of KFD in people ranges from 3 to 8 d, followed by sudden onset of the first clinical signs, such as chills, severe frontal headache, and fever of >40 °C. Other symptoms include bradycardia, generalized myalgia, severe prostration, conjunctival suffusion, and photophobia. Cervical and axillary lymph nodes are often enlarged, and virtually all patients have papulovesicular lesions in the soft palate^2,6^. Gastrointestinal symptoms and vomiting are also common. Hemorrhagic symptoms develop only in some of those infected and include gastrointestinal bleeding with hematemesis and/or blood in the stool. Bleeding from the nose and gums also has been reported^7^. In most patients, the infection has two phases, with an initial symptomless period lasting 1–2 weeks. Neurological symptoms dominate the second phase of the disease, and pulmonary edema is described as the main cause of death in fatal cases^8–10^. Despite the high medical importance of KFD and its alarming epidemiological activity in recent years, its pathogenesis remains largely unstudied^11^. Research on KFD pathogenesis is complicated by the fact that rodent models do not recapitulate human disease; mice infected with KFDV do not exhibit liver and spleen pathology or hemorrhagic signs seen in humans and develop a lethal neuroinfection^11,12^.

Endothelial cells (ECs) are thought to play a critical role in the pathogenesis of viral hemorrhagic fevers (VHFs). Viral infection–induced hemorrhage, increased vascular permeability, and/or acute edema can arise by following three major mechanisms: (i) the virus infects ECs, activating them (direct mechanism); (ii) the virus infects immune cells, which release soluble factors, activating ECs (indirect mechanism); and (iii) a combination of both direct and indirect mechanisms^13–15^. EC activation is associated not only with increased vascular permeability but also with the initiation of inflammatory responses and recruitment of leukocytes by the upregulation of leukocyte adhesion molecules, such as intercellular adhesion molecule 1 (ICAM1; also known as CD54), vascular cell adhesion molecule 1 (VCAM1; also known as vascular cell adhesion protein 1 or CD106), and E-selectin (also known as CD62 antigen-like family member E or CD62E), endothelial-leukocyte adhesion molecule 1, and leukocyte-endothelial cell adhesion molecule 2^15,16^. Several hemorrhagic fever-associated viruses also target dendritic cells (DCs), and these cells represent an important source of cytokines, which contribute to the development of hemorrhagic symptoms^17–19^.

In this study, we investigated the interaction of KFDV with human primary ECs and human monocyte-derived (mo) DCs under in vitro conditions to determine the target cell populations for KFDV infection and investigate the mechanism underlying the development of hemorrhagic complication during KFD. To the best of our knowledge, this study reports for the first time that human ECs are permissive to KFDV infection, which leads to EC activation, release of interleukin (IL)-6, a marked increase in leukocyte adhesion molecule expression, and increased leukocyte adhesion. MoDCs are also permissive to KFDV infection, which induces cytokine release. Treatment of ECs with culture supernatants from infected moDCs results in increased leukocyte adhesion to the ECs. The results indicate that both direct and indirect mechanisms can contribute to EC activation during KFD.

## Results

### KFDV can infect and replicate in human vascular ECs

**Fig. 1:**
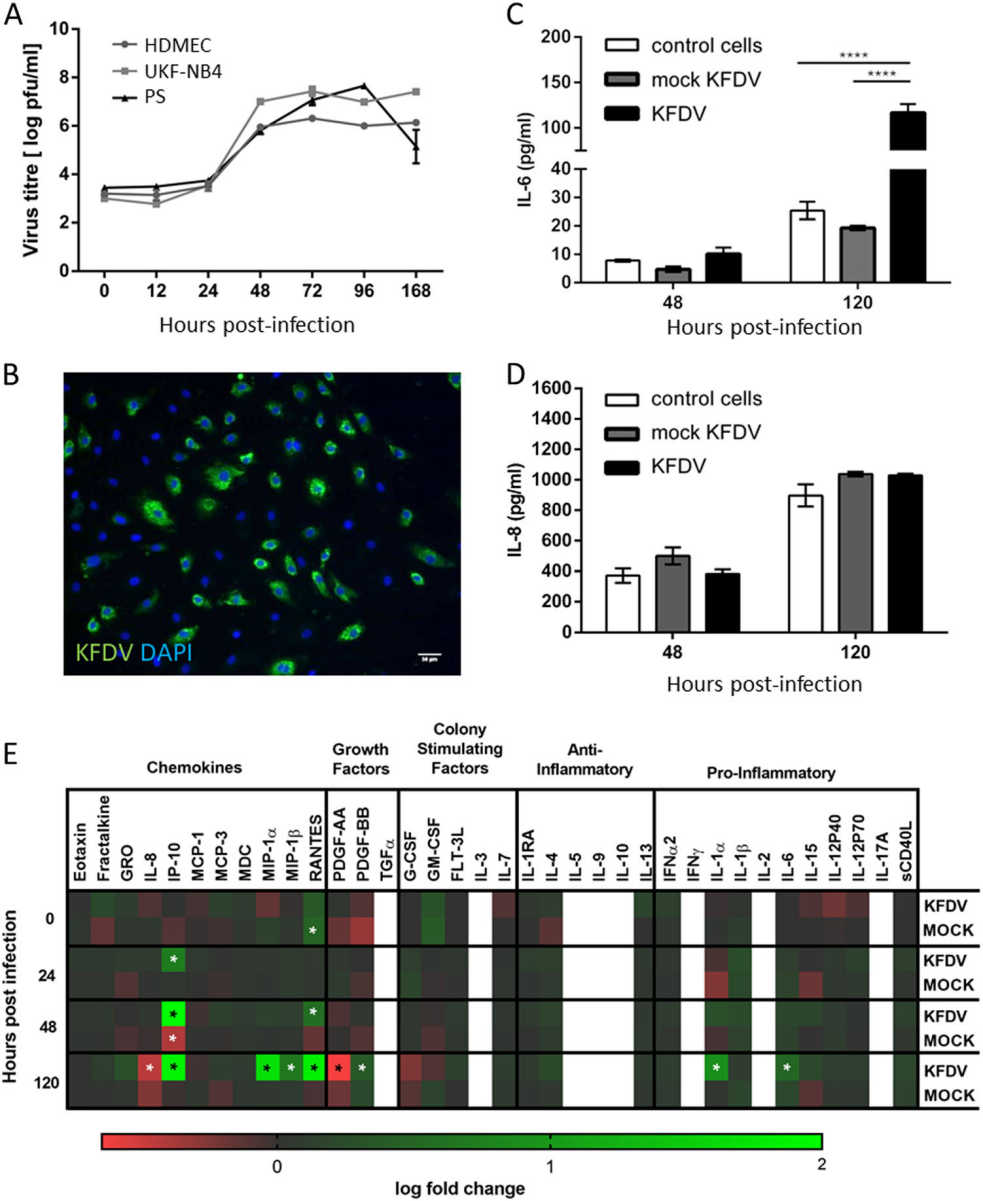
KFDV can infect and replicate in primary HDMECs, leading to increased IL-6 production. **a** HDMECs, PS, and UKF-NB4 cells were infected with KFDV at an MOI of 10. At the indicated time points, supernatants from the infected cells were collected, and KFDV titers were measured by plaque assay. **b** KFDV-infected and mock-infected HDMECs grown on slides were fixed at 48 h p.i. and immunostained with flavivirus E protein–recognizing antibody (green) and counterstained with DAPI (blue). **c**, **d** Supernatants from KFDV-infected, uninfected and mock-infected (UV-inactivated virus) HDMECs were collected at 48 and 120 h p.i., and levels of IL-6 and IL-8 were determined by ELISA. Data represent the mean concentrations ± SEM. ***p < 0.001. **e** Supernatants from KFDV-infected, uninfected and mock-infected (UV-inactivated virus) HDMECs were collected at 0, 24, 48 and 120 h p.i., and levels of multiple cytokines, chemokines and growth factors were determined by the Milliplex 41-plex human cytokine/chemokine magnetic bead panel assay. “Discoveries” as described in the Material and Methods are marked by an asterisk

KFDV infection and replication kinetics in HDMECs were determined by plaque assay and by immunofluorescence staining for viral antigen. KFDV replication was quantified using KFDV-infected cell supernatants collected at 0, 12, 24, 48, 72, 96, and 168 h p.i. at MOI = 10. Productive KFDV replication was first detected at 48 h p.i., and this interval also represented a peak for virus production. Specifically, there was an increase in the KFDV titer of approximately three logs between 24 and 48 h p.i. Viral titers remained approximately at the same level from 48 h p.i. up to the end of the experiment (Fig. 1a). Immunofluorescence staining for viral envelope antigen performed at 48 h p.i. revealed that almost 70% of cells in HDMEC culture were infected (Fig. 1b).

KFDV growth kinetics in HDMECs were compared with virus growth curves in PS and UKF-NB4 cells, which are other cell lines known to replicate flaviviruses. PS and UKF-NB4 cells were infected with KFDV, and cell culture supernatants collected at the same time points used in the experiment with HDMECs; viral titers were determined using the plaque assay. Similar KFDV growth curves in PS and UKF-NB4 cultures were observed relative to the viral growth curve in HDMECs (Fig. 1a), but maximal viral titers were approximately 10 times higher in PS and UKF-NB4 cells than in HDMECs. Only at 168 h p.i., the KFDV titer in PS cell culture dropped rapidly, probably because of a gross cytopathic effect that was not seen in HDMEC culture (Fig. 1a).

### KFDV activates the release of IL-6 but not IL-8 in infected ECs

We used ELISA to investigate the release of IL-6 and IL-8 in the culture medium of KFDV or mock-infected HDMECs at 48 and 120 h p.i. Mock-infected HDMECs were exposed to the same dose of UV-inactivated KFDV as HDMECs infected with viable virus. Mock-infected HDMECs showed weak production of IL-6, which was higher in cells infected with viable KFDV at 48 h (*p* < 0.05) and more markedly at 120 h (*p* < 0.001) p.i. Background expression of IL-8 in resting HDMECs was high, but no significant differences were observed in the production of IL-8 between the mock- and KFDV-infected cells (Fig. 1c, d).

A Milliplex 41-plex human cytokine/chemokine magnetic bead panel was used to measure changes in the release of 41 human cytokines, chemokines and growth factors in the culture medium of KFDV- or mock-infected HDMECs at 0, 24, 48 and 120 h p.i. This analysis provided further evidence that IL-6 expression and release is increased in response to KFDV infection (Fig. 1e). While ELISA analysis did not show any changes in the expression of IL-8, the Milliplex assay indicated a slight downregulation of IL-8 production at 120 h p.i. (Fig. 1e). Several other chemokines, such as interferon gamma-induced protein 10 (IP-10, also known as C-X-C motif chemokine 10, CXCL10), RANTES (regulated on activation, normal T cell expressed and secreted; also known as chemokine (C-C motif) ligand 5, CCL5), macrophage inflammatory protein 1-alpha (MIP-1-alpha; also known as CCL3), and MIP-1-beta (also known as CCL4), were strongly upregulated by HDMECs in response to KFDV infection. Additionally, expression of IL-1α and platelet-derived growth factor (PDGF-BB) was significantly increased, while expression of PDGF-AA was downregulated in KFDV-infected HDMECs at 120 h p.i. Levels of transforming growth factor alpha (TGFα), IL-2, IL-3, IL-5, IL-9, IL-10, and IFNγ were below the detection limit. Epidermal growth factor (EGF), basic fibroblast growth factor (FGF-2), and vascular endothelial growth factor (VEGF) were excluded from the analysis because of high levels of these analytes are present in cell culture supernatants and originate from culture supplements used for HDMEC cultivation.

### KFDV infection upregulates the expression of adhesive molecules in ECs

**Fig. 2:**
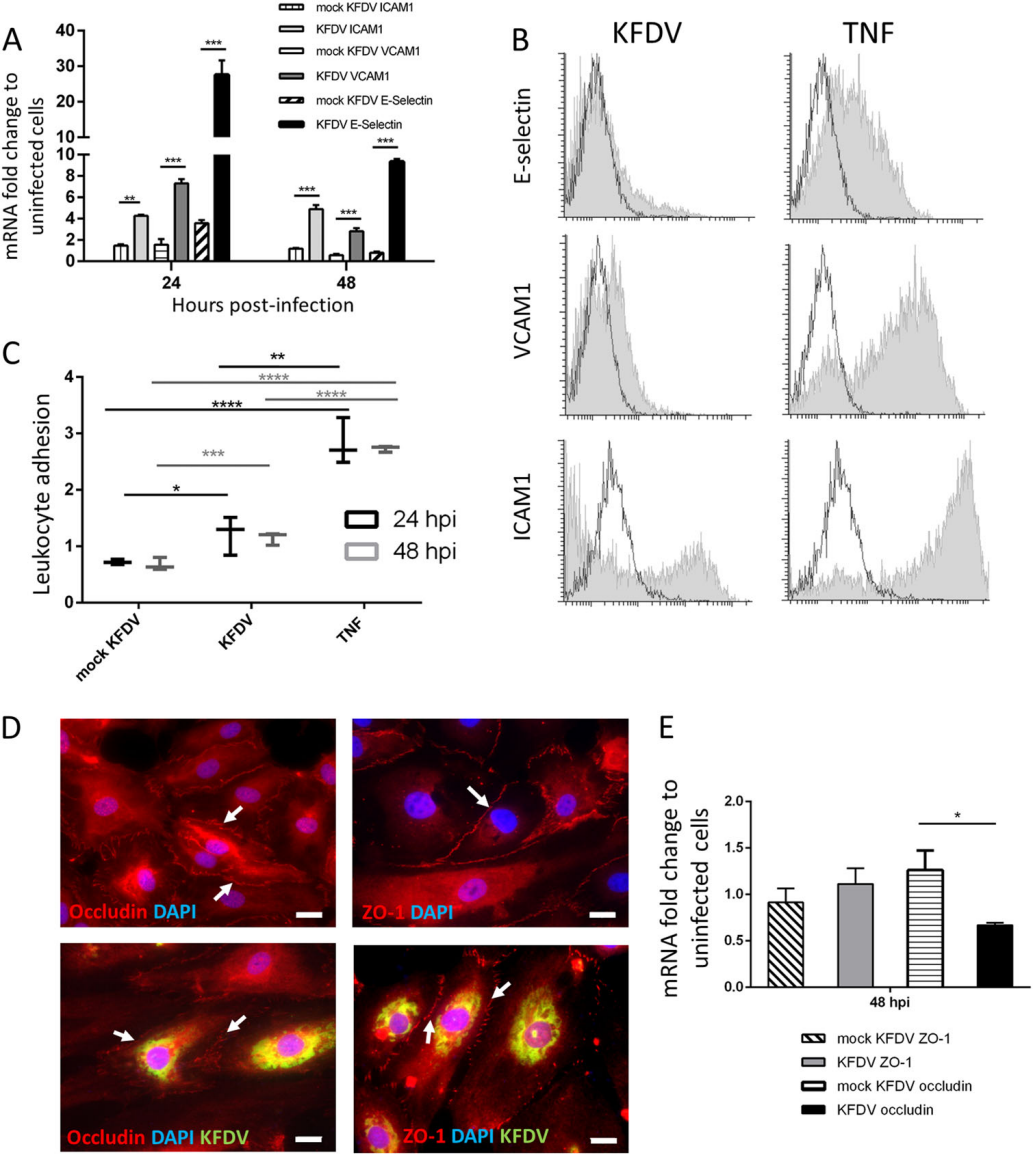
KFDV infection of HDMECs leads to their activation. **a** HDMECs were infected with KFDV at an MOI of 10. Mock-infected HDMECs were exposed to the same dose of UV-inactivated KFDV. At 24 and 48 h p.i., mRNA expression of E-selectin, ICAM1, and VCAM1 was analyzed by qRT-PCR and normalized to the expression of housekeeping genes. Fold increase indicates differences in normalized mRNA expression relative to expression in noninfected cells. **b** Flow cytometry was used to measure the protein levels of E-selectin, VCAM1, and ICAM1 expression in the KFDV-infected HDMECs at 48 h p.i. The production of cell-adhesion molecules is shown in overlapping histograms of relative fluoresce intensity in the analyzed cells; empty histograms with solid lines represent mock-infected (UV-inactivated virus) HDMECs, and gray histograms represent the KFDV-infected cells. **c** HDMECs were treated with TNF-α (positive control) or mock-infected with UV-inactivated KFDV or infected with KFDV at an MOI of 10 and incubated for 24 and 48 h. After incubation, the adhesion of BCECF–stained leukocytes was measured as described in the Materials and Methods. The leukocyte adhesion index represents the fold change relative to controls (y-axis). **d** HDMECs grown and fixed on slides at 48 h p.i. were stained with anti-flavivirus envelope (green) and anti-occludin (red) or anti-ZO-1 antibodies and counterstained with DAPI (blue). Bar = 20 µm. **e** Total RNA from uninfected, mock- and KFDV-infected HDMECs 48 h p.i. was used to determine the fold change in occludin and ZO-1 mRNA expression by qRT-PCR. *p < 0.05; **p < 0.01; ***p < 0.001; ****p < 0.0001. h.p.i. = hours post infection

The effect of KFDV infection on the expression of key cell matrix adhesion molecules (CAMs), proteins located on the cell surface and involved in adhesive interactions with leukocytes, was investigated by qRT-PCR. HDMECs were infected with KFDV or mock-infected with UV-inactivated KFDV, and the expression of ICAM1, VCAM1, and E-selectin mRNA was measured at 24 and 48 h p.i. The expression was normalized to mRNA levels of two housekeeping genes and compared to the expression in mock-infected cells. At 24 h p.i., mRNA levels of ICAM1 (*p* < 0.01), VCAM1 (*p* < 0.001), and E-selectin (*p* < 0.001) in KFDV-infected cells were highly upregulated and significantly higher than those in cells stimulated with inactivated virus (Fig. 2a). At 48 h p.i., the mRNA levels of VCAM1 and E-selectin in KFDV-infected HDMECs were slightly lower than at 24 h p.i., but the mRNA levels of all measured CAMs were still significantly higher in KFDV-infected cells than in mock-infected cells (*p* < 0.001) (Fig. 2a).

Flow cytometry was used to measure the protein level of CAM expression in KFDV-infected HDMECs at 48 h p.i. Unstimulated HDMECs expressed low levels of ICAM1 and hardly any VCAM1 and E-selectin (Fig. 2b, empty lines). Treatment with TNF-α for 48 h (positive control) led to strong production of ICAM1 and VCAM1 and mild expression of E-selectin. After KFDV infection of the cells (MOI 10), ICAM1 was most noticeably expressed (with 20.0% ICAM1–positive cells), whereas E-selectin and VCAM1 production were not so prominent (10.7 and 9.8% positive cells, respectively) (Fig. 2b).

### KFDV infection of ECs increases leukocyte adhesion

HDMECs were treated with TNF-α (positive control), mock-infected with UV-inactivated KFDV, or infected with KFDV at MOI 10 and incubated for 24 and 48 h. After incubation, the adhesion of BCECF–stained leukocytes was measured as described in the “Materials and Methods” section. The level of leukocyte adhesion was normalized against the leukocyte adhesion in untreated and uninfected HDMECs. Treatment of HDMECs with TNF-α increased leukocyte adhesion by these cells substantially (Fig. 2c). Additionally, KFDV infection induced a significant increase in leukocyte adhesion by HDMECs at 24 (*p* < 0.05) as well as 48 h (*p* < 0.001) p.i., while stimulation with inactivated virus did not impact leukocyte adhesion.

### KFDV infection does not alter ZO-1 but downregulates occludin expression in HDMECs

KFDV-infected HDMEC monolayers were investigated for alterations in the expression and distribution of occludin and ZO-1, key tight junction (TJ) membrane proteins, by measurement of mRNA production and immunofluorescent staining. The cells were fixed and stained at 48 h p.i. KFDV-infected cells were identified by immunofluorescent staining of the viral envelope antigen. Distinct ZO-1 staining was observed in uninfected cells, but the staining was slightly fragmented in KFDV-infected cells relative to the controls (Fig. 2d). In KFDV-infected cells, occludin immunoreactivity was substantially reduced relative to control cells (Fig. 2d). Changes in the levels of occludin and ZO-1 mRNA were normalized to the expression of housekeeping gene, and then the fold change in the infected cells was determined relative to corresponding controls. Expression of ZO-1 mRNA was similar in both mock- and KFDV-infected cells, but the expression of occludin mRNA was significantly reduced in KFDV-infected cells (*p* < 0.05) (Fig. 2e).

### MoDCs are permissive to KFDV infection

**Fig. 3:**
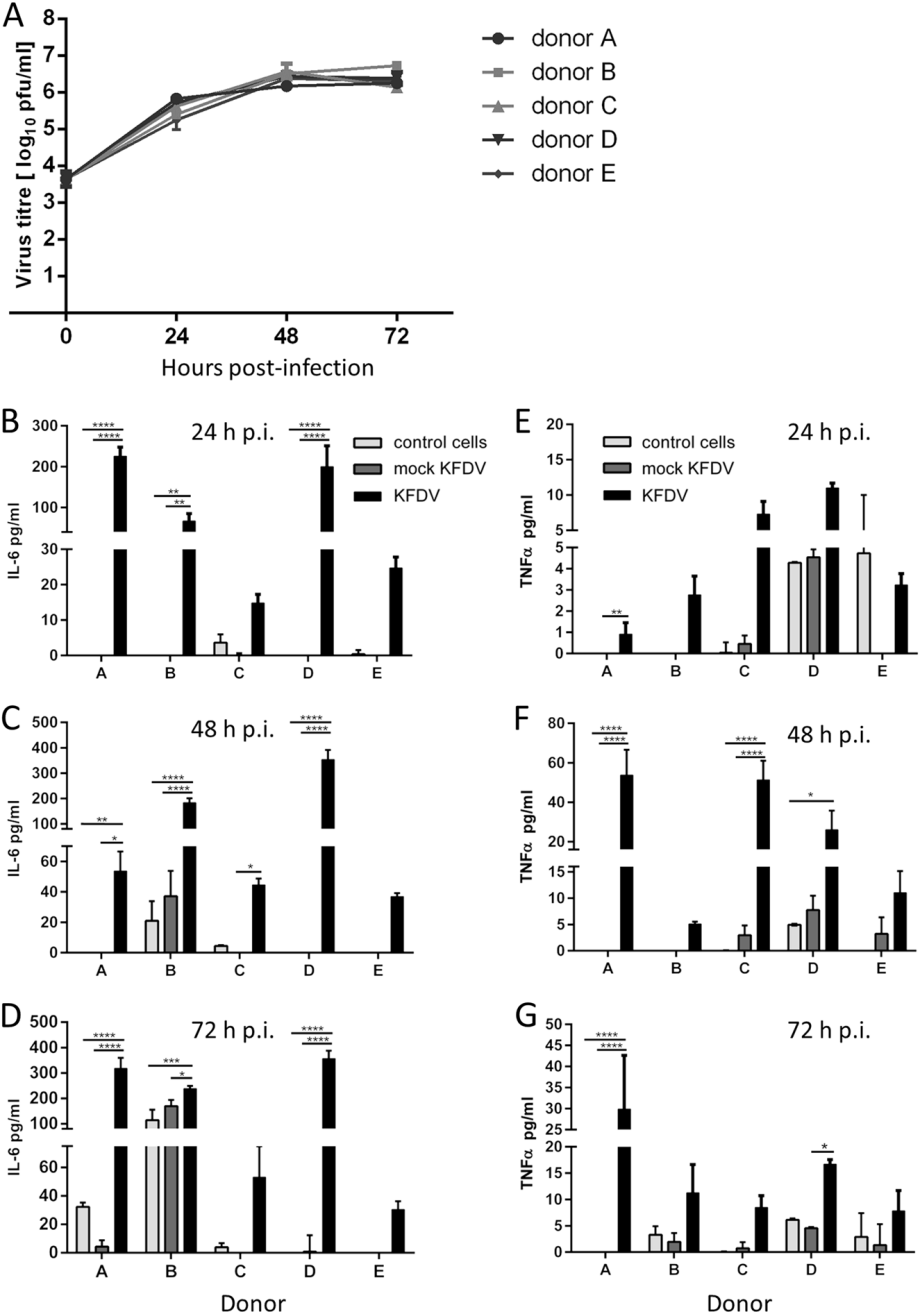
KFDV can infect and replicate in human moDCs, leading to increased IL-6 and TNF-α production. **a** PBMCs were obtained from buffy coats from 5 healthy and naïve donors (**a**–**e**). Monocytes were purified from PBMCs by positive magnetic separation. MoDCs were obtained and infected with KFDV as described in the Materials and Methods, and viral growth was quantified in cell culture supernatants at 0, 24, 48, and 72 h p.i. using the plaque assay. **b**–**d** Supernatants from uninfected (control), KFDV-infected and mock-infected (UV-inactivated virus) moDCs were collected at 24 (**b**), 48 (**c**), and 72 (**d**) h p.i., and levels of IL-6 were determined by ELISA. **e**–**g** Supernatants from KFDV-infected and mock-infected (UV-inactivated virus) moDCs were collected at 24 (**b**), 48 (**c**), and 72 (**d**) h.p.i., and levels of TNF-α were determined by ELISA. Data represent mean concentrations ± SEM. *p < 0.05; **p < 0.01; ***p < 0.001; ****p < 0.0001

PBMCs were obtained from buffy coats from five healthy and naïve donors (termed A, B, C, D, and E; all were TBEV antibody-negative). Monocytes were purified from PBMCs by positive magnetic separation. MoDCs were obtained following 6-d culture with IL-4 and granulocyte-macrophage colony-stimulating factor. MoDCs were infected with KFDV as described in the Materials and Methods, and viral growth was quantified in cell culture supernatants at 0, 24, 48, and 72 h p.i. using the plaque assay. Productive KFDV replication in the form of released virions was detected early, at 24 h p.i. Within the first 24 h p.i., the titer in the cell culture supernatants increased more than 100-fold. From 24 to 72 h p.i., the viral titer in the culture medium increased further by approximately 10-fold (Fig. 3a). Similar viral growth kinetics and maximal viral titers were observed in moDCs derived from PBMCs obtained from each of the five donors (Fig. 3a).

### KFDV infection of moDCs activates the cytokine response

MoDCs derived from monocytes obtained from five donors were infected with KFDV or mock-infected with UV-inactivated KFDV, and cell-free supernatants were collected at 24, 48, and 72 h p.i. and analyzed by ELISA for IL-6 and TNF-α concentrations. A certain degree of interindividual variability in cytokine production by KFDV-infected moDCs originating from different donors was observed (Fig. 3b–g). Substantially higher levels of IL-6 were measured in supernatants from KFDV-infected moDCs relative to mock-infected or uninfected cells at 24 and 48 h p.i. (Fig. 3b, c). At 72 h p.i., increased levels of IL-6 were seen in KFDV-infected moDCs derived only from some donors (Fig. 3d). MoDCs derived from Donor B also produced high levels of IL-6 after mock infection. TNF-α production by KFDV-infected moDCs was seen in cells derived from most donors. The highest levels of TNF-α production were observed at 48 h p.i. (Fig. 3e–g).

### Supernatants from KFDV-infected moDCs activate ECs

**Fig. 4:**
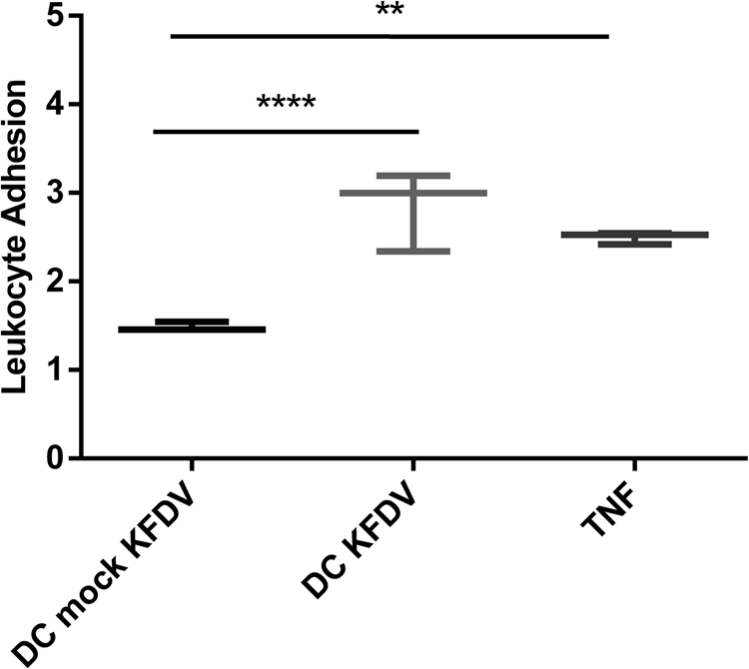
Supernatants from KFDV-infected moDCs activate ECs. Supernatants from the moDCs infected with KFDV or mock-infected with UV-inactivated virus for 48 h were collected and incubated with monolayers of HDMECs for 6 h. After incubation, the leukocyte adhesion assay was performed as described in the Materials and Methods. HDMECs treated with TNF-α were used as a positive control. The level of leukocyte adhesion was normalized against leukocyte adhesion in untreated and uninfected HDMECs and is expressed as the leukocyte adhesion index representing the fold change relative to controls. The data are represented by a boxplot with minimal and maximal values. **p < 0.01; ****p < 0.0001

To investigate if supernatants from KFDV-infected moDCs can activate HDMECs and increase leukocyte adhesion, supernatants from the moDCs infected with KFDV or mock-infected with UV-inactivated virus for 48 h were collected and incubated with monolayers of HDMECs for 6 h. After incubation, a leukocyte adhesion assay was performed as described in the “Materials and Methods” section. HDMECs treated with TNF-α were used as a positive control, and data were normalized against uninfected cells. Data presented in Fig. 4 show that treatment of HDMECs with supernatants from KFDV-infected moDCs caused their activation, as demonstrated by an increased leukocyte adhesion index (*p* < 0.001) relative to HDMECs treated with supernatants from mock-infected moDCs (Fig. 4).

## Discussion

While most tick-borne flaviviruses pathogenic for humans cause encephalitis of varying severity, KFDV together with Alkhurma (Alkhumra) virus and Omsk hemorrhagic fever virus can cause hemorrhagic manifestations^5,20^. However, not all patients infected with KFDV develop hemorrhagic symptoms, which could possibly be influenced by the virulence of the particular virus strain or by host age and immune and overall health status. Research on several other viral hemorrhagic fevers, such as Crimean-Congo hemorrhagic fever (CCHF), hantaviral or filoviral infections, and dengue, indicates that vascular ECs play a crucial role in the development of hemorrhagic complications. The mechanism of the hemorrhagic manifestations observed during KFD remains completely unknown. The viruses activate the ECs, which results in vascular pathology. Hemorrhagic manifestations arise from inter-EC gaps caused by physical damage to the endothelium that permit both fluid and cellular effluxes into the tissue^14^. EC activation may result from direct viral infection of these cells, an indirect effect of soluble mediators produced by infected immunocompetent cells, or an interplay involving both. This study is the first, to the best of our knowledge, to demonstrate that both ECs and moDCs are permissive to KFDV infection, which leads to EC activation by both direct and indirect mechanisms.

KFDV replicated in ECs with kinetics similar to those observed for PS or UKF-NB4 cells, reaching maximum titers of approximately 6 log_10_ plaque-forming units/ml, which was, however, 10 times lower than the maximal titers observed in KFDV-infected cultures of PS and UKF-NB4 cells. The infection of PS and UKF-NB4 cells was accompanied by a gross cytopathic effect that was not seen in the culture of infected ECs. Only a few hemorrhagic fever-associated viruses, such as hantaviruses or CCHF, predominantly infect human ECs, suggesting a direct effect of viral infection on EC function that contributes to the pathology^15,21–23^. ECs are probably not a primary target for KFDV, but their infection can occur during viremia in the early stages.

KFDV infection of ECs increased the production of IL-6 by these cells, similar to results from a study investigating the infection of ECs by CCHF virus^15^. IL-6 has dual roles as both an anti-inflammatory and proinflammatory mediator, and its serum levels are enhanced in patients with hemorrhagic fevers of various viral origins, such as dengue hemorrhagic fever/shock syndrome, CCHF, Hantaan virus infection in patients with hemorrhagic fever with renal syndrome, and Ebola^24–28^.

CCHFV also increases the expression of IL-8 in infected ECs^15^, which was not observed in KFDV-infected ECs in the current work. IL-8 is a potential chemoattractant and increases endothelial adherence, extravasation of leukocytes, and migration of these cells into tissues^13,29–31^. Elevated serum levels of IL-8 correlate with the severity of hemorrhagic manifestations, such as during dengue hemorrhagic fever^32^, CCHF, and Ebola^28,33^. On the other hand, infection of ECs with Lassa virus leads to the suppression of IL-8 expression at both the protein and mRNA levels^29^. The present study indicates that ECs infected with KFDV do not contribute to the development of hemorrhagic symptoms via increased production of IL-8.

In addition to IL-6, KFDV infection of ECs increased the expression of IL-1α, IP-10, RANTES, MIP-1α, and MIP-1β. While these cytokines/chemokines can be important in establishing the early host response to infection, aberrant or dysregulated expression can have detrimental effects contributing to the development of the health complications seen during infection^28,34^.

This work also showed that ECs infected with KFDV increase CAM expression, in particular ICAM1, which leads to increased leukocyte adhesion to ECs. ICAM1 is expressed at only low levels by unstimulated ECs, but exposure to proinflammatory cytokines or infection can upregulate its expression^15,35^. ICAM1 is a ligand for LFA-1 (integrin), a receptor found on leukocytes^36^. When the expression of ICAM1 is activated, leukocytes bind to the ECs via ICAM1/LFA-1 and then transmigrate into the tissue to a site of inflammation or injury^37^. Results similar to ours, demonstrating EC activation, increased ICAM1 expression, and enhanced leukocyte adhesion, have been obtained when ECs were infected with various other viruses^15,38–42^.

Tight junction (TJ) proteins play a critical role in vascular homeostasis. Here, we investigated the effect of KFDV infection on two important TJ proteins, occludin and ZO-1, in ECs. KFDV had no effect on the mRNA expression of ZO-1, but the localization of ZO-1 on cell–cell boundaries was slightly fragmented relative to controls. Expression of occludin was significantly reduced after infection with a correlating decrease in occluding RNA levels. Research on other VHFs indicated that infection can lead to the redistribution of TJ proteins, increasing paracellular permeability. This is believed to be caused by the release of cytokines from infected cells rather than by the virus itself^31,43,44^. In the case of RVFV, however, no effect on TJ proteins is seen in infected EC culture^45^. Further studies need to address whether the downregulation of TJ expression or TJ degradation is the main cause of hemorrhagic signs seen in KFD patients.

Systemic release of proinflammatory mediators is a key hallmark of viral hemorrhagic fevers^46–48^. DCs are one of the potential sources of inflammatory mediators during infection^49^, and these cells are targets for various viruses causing hemorrhagic fevers in humans, such as Hantaan orthohantavirus^19^, Ebola and Marburg filoviruses^50^, dengue virus^18^, CCHFV^17^, and Lassa virus^46^. However, infection of DCs by different viruses induces different cytokine responses. Ebola, Marburg, and Lassa viruses infect and replicate in human DCs without inducing cytokine secretion^46,50^. Infection of DCs by dengue virus, though, leads to cell maturation and production of TNF-α and interferon (IFN)-α, but not IL-6 and IL-12^18^. TNF-α and IFN-α production are also activated when DCs are infected with Hantaan virus^19^. Infection of DCs with Rift Valley Fever virus (RVFV) induces the expression of TNF-α, IL-6, and IL-10 but not IL-8, IL-19, or IL-1β^17^. Furthermore, supernatants from RVFV-infected DCs activate ECs and increase leukocyte adhesion^17^.

Our results show that KFDV represents another VHF-associated virus that readily infects and replicates in DCs. Similar to CCHFV, KFDV activated the expression of IL-6 and TNF-α in infected DCs. Supernatants from KFDV-infected cells in turn activated ECs, as documented by significantly increased leukocyte adhesion. Because the supernatants contained high amounts of TNF-α, this cytokine could be responsible for EC activation, as demonstrated in control ECs treated with TNF-α alone. More research on the precise mechanisms of EC activation is needed, but the results strongly indicate that ECs can be activated by both direct and indirect mechanisms during KFDV infection.

In summary, this study is the first, to the best of our knowledge, to show that KFDV can infect and replicate in ECs and DCs and that the activation of ECs during KFDV infection can presumably be caused by both direct and indirect mechanisms. One of the possible limitations of our study is the fact that we used HDMECs as model ECs because these cells could mimic aspects of the pathogenesis of petechia and rash seen in human KFD patients. However, most of the work on other VHFs has been done with ECs from other vascular beds, such as HUVECs, which could complicate the comparison of our work with other studies. Regardless, our findings are similar to previously published works with other VHFs demonstrating similar observations for several VHFs regardless of the EC origin. These data, in concert with work using other VHFs, suggest that some pathological mechanisms of KFD and other VHFs might be similar. KFD is a largely neglected infection with very few publications describing clinical and pathological findings in affected patients. Thus, putting our findings into a clinical context is difficult, and more extensive research in this field is needed to delineate in more detail the mechanisms underlying the hemorrhagic complications seen in some patients infected with KFDV.

## Materials and methods

### Biosafety statement

All work with KFDV was done in an accredited high-biocontainment facility at the Veterinary Research Institute in Brno and the Institute of Parasitology, Biology Centre of the Czech Academy of Sciences in Ceske Budejovice, Czech Republic. The use of KFDV in the experiments was approved by the Department of Biological Weapons Prohibition of the State Office for Nuclear Protection in Prague, Czech Republic (Permit No. SUJB/OKZBZ/15978/2013).

### Cells

Human dermal microvascular ECs (HDMECs) (Sciencell, Carlsbad, California) were cultured at 37 °C in fibronectin-coated flasks (Sciencell) containing complete EC medium (Sciencell). Porcine kidney stable (PS) cells were cultured at 37 °C in Leibovitz (L-15) medium supplemented with 3% fetal bovine serum, 100 U/ml penicillin, and 100 µg/ml streptomycin (Sigma-Aldrich, Czech Republic). Human neuroblastoma UKF-NB4 cells were cultured at 37 °C in 5% CO_2_ in Iscove’s modified Dulbecco’s medium supplemented with 10% fetal bovine serum and 292 µg/ml l-glutamine, 100 U/ml penicillin, and 100 µg/ml streptomycin (Sigma-Aldrich, Czech Republic).

Peripheral blood mononuclear cells (PBMCs) were obtained from five healthy blood donors (A–E) via the collection of buffy coats provided by the blood bank at the University Hospital in Brno and followed by Ficoll-Paque (GE Healthcare, UK) density gradient centrifugation. For positive selection, monocytes were incubated with anti-human CD14 microbeads (Miltenyi Biotec, Germany), followed by magnetic separation. The obtained monocytes were cultured for 6 d in RPMI 1640 at 37 °C (Sigma-Aldrich, Czech Republic) containing 100 ng/ml granulocyte-macrophage colony-stimulating factor (Peprotech, UK), 100 ng/ml IL-4 (Peprotech, UK), 10% fetal bovine serum, and 2 mM L-glutamine (Sigma-Aldrich, Czech Republic) for DC differentiation. DC purity was verified by staining for DC-SIGN (CD209) (Exbio, Czech Republic). Flow cytometry was performed using an LSR Fortessa flow cytometer (BD Biosciences) operated by Diva software (Becton Dickinson).

### Virus

The KFDV strain W-377 was used in all experiments (GenBank Accession No. JF416960). The virus was isolated from *Semnopithecus entellus* in 1957 in Karnataka, India (Work et al., 1957) and is widely used as a prototype representative of KFDV. The virus was provided by the Collection of Arboviruses, Institute of Parasitology, Biology Centre of the Czech Academy of Sciences, Ceske Budejovice, Czech Republic (http://www.arboviruscollection.cz/index.php?lang=en). Before its use in experiments, the virus was passaged multiple times in suckling mouse brains and once in UKF-NB4 cells. The supernatants were used as the viral stock (10^6^ plaque-forming units/ml). The virus titer was determined by plaque assay as described below^51^.

To generate ultraviolet (UV)-inactivated KFDV, a viral suspension was exposed to UV light while on ice for 1 h using a UV Crosslinker CL-508 (Uvitec Cambridge). Inactivation of the virus infectivity was verified by plaque assay.

### Viral growth assay

Confluent HDMEC, PS, and UKF-NB4 cells grown in 96-well plates were infected with KFDV at a multiplicity of infection (MOI) of 10. After 3 h, unattached virus was removed by three washing steps. The cells were incubated at 37 °C in 5% CO_2_ (HDMEC and UKF-NB4) or at 37 °C and atmospheric CO_2_ concentration (PS). At 12 h post infection (p.i.) and 1, 2, 3, 4, 5, and 7 d p.i., supernatant medium from appropriate wells was collected and frozen at −70 °C. Virus titers were determined by plaque assay.

MoDCs were infected with KFDV at an MOI of 10 and incubated at 37 °C in 5% CO_2_ and a humidified atmosphere. The supernatants were collected at 0, 24, 48, or 72 h p.i. and frozen at −70 °C. Titers were determined by plaque assay.

### Plaque assay

Virus titers were assayed on PS cell monolayers, as described previously^51^. Briefly, 10-fold dilutions of KFDV supernatants from infected cells were prepared in 24-well tissue culture plates, and PS cells were added in suspension (1.2 × 10^5^ cells per well). After a 4-h incubation, the suspension was overlaid with 1.5% (wt/vol) carboxymethylcellulose in L-15 medium. Following incubation for 5–6 d at 37 °C, the infected plates were washed with phosphate-buffered saline (PBS), and the cell monolayers were stained with naphthalene black. The virus titer was expressed as plaque-forming units per milliliter.

### Immunofluorescence staining

Infected and noninfected HDMECs grown on slides were subjected to 4% formaldehyde fixation for 1 h, rinsed in PBS + 0.05% Tween 20, permeabilized with 0.2% Triton X-100, and blocked with 5% goat serum. Cells were labeled with a flavivirus-specific monoclonal antibody (clone D1-4G2-4-15; 1:250; MilliPore) for 1 h at 37 °C. After washes with Tween 20 (0.05 %, v/v) in PBS, the cells were labeled with an anti-mouse goat secondary antibody conjugated with fluorescein isothiocyanate (FITC) (1:500; Sigma-Aldrich) for 1 h at 37 °C. Staining of key tight junction proteins was done with a rabbit anti-occludin (1: 14, Invitrogen) or rabbit anti-ZO-1 antibody (1: 200, Invitrogen) for 1 h at 37 °C. The cells were counterstained with 4’,6-diamidino-2-phenylindole (DAPI) (1 µg/ml; Sigma-Aldrich, Czech Republic) for 30 min at 37 °C, mounted in 2.5 % 1,4-diazabicyclo(2.2.2)octane (DABCO), an anti-fade reagent (Sigma-Aldrich, Czech Republic), and examined with an Olympus IX71 epifluorescence microscope equipped with a Hamamatsu OrcaR2 camera and controlled using Xcellence software. The images were processed using Fiji software^52^.

### Quantitative real-time RT-PCR

Changes in gene expression in KFDV-infected HDMECs were measured by quantitative real-time (qRT)-PCR. Monolayer HDMEC cultures grown in 96-well plates were infected with KFDV at an MOI of 10. After 3 h at 37 °C and 5% CO_2_, the cells were washed and further cultured in fresh growth medium. Mock-infected control HDMECs were infected with UV-inactivated KFDV. KFDV-infected and mock-infected HDMECs and control cells were lysed at 24 and 48 h p.i., and cDNA was synthesized by reverse transcription using an Ambion Cells-to-CT kit (Applied Biosystems, Thermo Fisher Scientific Inc, UK) according to the manufacturer’s instructions. The synthesized cDNAs were used as templates for qRT-PCR. PCR was performed using predeveloped TaqMan Assay Reagents [Assay IDs: ICAM-1 (Hs00164932_m1), VCAM-1 (Hs01003372_m1), SELE (Hs00174057_m1), occludin (Hs00170162_m1), and zonula occludens-1 (ZO-1/TJP1; Hs01551876_m1)] and TaqMan Gene Expression Master Mix (Applied Biosystems) on a LightCycler 480 II with a 96-well plate block (Roche, UK). Human β-actin (Hs99999903_m1) and glyceraldehyde 3-dehydrogenase (Hs03929097_g1) were used as housekeeping genes. The amplification conditions were as follows: 2 min at 50 °C (to allow uracil-*N*-glycosylase to destroy any contaminating templates); 10 min at 95 °C (to denature UNG and activate the enzymes); 40 cycles of denaturation at 95 °C for 15 s; and annealing/extension at 60 °C for 1 min.

To calculate the fold change in gene expression, the cycle threshold (CT) of the housekeeping genes was subtracted from the CT of the target gene to yield ΔCT. Change in the expression of the normalized target gene was expressed as 2^–ΔΔCT^, where ΔΔCT = ΔCT sample−ΔCT control, as described previously^53^.

### Enzyme-linked immunosorbent assay

Cytokine production by the KFDV-infected HDMECs and moDCs was measured by enzyme-linked immunosorbent assay (ELISA). Monolayer HDMEC cultures grown in 96-well plates were infected with KFDV at an MOI of 10. After 3 h at 37 °C and 5% CO_2_, the cells were washed and further cultured in fresh growth medium. Mock-infected control HDMECs were infected with UV-inactivated KFDV. Supernatants from infected, uninfected, and mock-infected HDMECs were collected at 48 and 120 h p.i. and assayed for levels of IL-6 and IL-8 using ELISA according to the manufacturer’s recommendations (eBioscience, Inc., San Diego, CA).

MoDCs were infected with KFDV at an MOI of 10 or UV-inactivated (mock) KFDV and incubated at 37 °C in 5% CO_2_ and a humidified atmosphere for 24, 48, or 72 h p.i. The supernatants were collected and assayed for levels of IL-6 and tumor necrosis factor (TNF)-α by ELISA (eBioscience, Inc.) according to the manufacturer’s protocol.

### Multiplex cytokine bead array assay

Monolayer HDMEC cultures grown in 96-well plates were infected with KFDV at an MOI of 10 or UV-inactivated KFDV (mock). After 3 h of incubation at 37 °C and 5% CO_2_, the cells were washed and further cultured in fresh growth medium. Cytokine and chemokine levels in medium supernatant collected at 0, 24, 48, and 120 h p.i. were determined using the Milliplex 41-plex human cytokine/chemokine magnetic bead panel (HCYTMAG-60K-PX41; Millipore) and a MAGPIX instrument (Luminex, Austin, TX), according to the manufacturer’s instructions. Data were collected using xPonent software (Luminex), log-transformed, and analyzed as described in the Statistical analysis section.

### Flow cytometry determination of E-selectin, VCAM1, and ICAM1 expression in KFDV-infected HDMECs

HDMECs were seeded on a 6-well plate at a concentration of 1 × 10^5^ cells per ml and infected by KFDV at an MOI of 10. As a positive control, HDMECs were treated with TNF-α (10 ng/ml). After 48 h, cells were washed with Dulbecco’s PBS (Sigma), harvested in 0.2% EDTA (Sigma), and labeled for 20 min at 4 °C with the following antibodies: anti-human CD62E-PE (E-selectin), anti-human CD54-APC (ICAM-1), and anti-human CD106-PE-Cy7 (VCAM-1) (all from eBioscience). The cells then were washed with 1% fetal bovine serum in PBS, fixed with fresh 2% formaldehyde solution, washed again with 1% fetal bovine serum in PBS, and measured by flow cytometry using BD FACS CANTO II with BD FACS Diva software. Data were further analyzed using Flowing Software 2, version 2.5.1 (Perttu Terho, University of Turku, Finland).

### Leukocyte adhesion assay

The method for the measurement of leukocyte adhesion to ECs under static conditions has been described previously^54^. Briefly, HDMEC layers were treated with TNF-α (10 ng/ml) as a positive control, infected with KFDV or UV-inactivated (mock) KFDV at an MOI of 10, and incubated for 24 and 48 h p.i. Leukocytes were isolated from five human buffy coats (marked as A–E; provided by the University Hospital in Brno) by Ficoll-Paque (GE Healthcare) density gradient centrifugation as described previously and stained with 2 µM 2‘,7“-bis-(2-carboxyethyl)-5(6)-carboxyfluorescein acetoxymethyl ester in Hanks balanced salt solution containing 25 mM HEPES (Invitrogen Life Technologies, UK)^17^. The leukocytes were finally suspended in RPMI 1640 medium containing 10% fetal bovine serum, 100 U/ml penicillin, and 100 µg/ml streptomycin (Sigma-Aldrich, Czech Republic).

HDMEC monolayers were washed extensively with fresh medium and cocultured with 2‘,7“-bis-(2-carboxyethyl)-5(6)-carboxyfluorescein acetoxymethyl ester (BCECF)–stained leukocytes for 1 h at 37 °C in 5% CO_2_. Nonadherent leukocytes were washed away with PBS. Lysis buffer (50 mM Tris-HCl, 0.1% sodium dodecyl sulfate in water, pH 8.2–8.4) was added to the remaining cells. After cell lysis, the fluorescence of BCECF was detected by exciting the lysates at 485 nm, and emission was read at 528 nm on a Synergy HT microplate reader (Bio-Tek, Winooski, Vermont). Data were collected and analyzed using Gen5 software (Bio-Tek).

MoDCs (donor D) were infected with KFDV at an MOI of 10 or with UV-inactivated (mock) KFDV and incubated at 37 °C in a 5% CO_2_ humidified atmosphere for 48 h p.i. The supernatants were collected and saved for further analysis. Confluent HDMECs were exposed to the collected (UV-inactivated) supernatants from moDCs for 6 h, washed with fresh medium, and subsequently subjected to the leukocyte adhesion assay as described above.

### Statistical analysis

The data in graphs are expressed as the mean ± standard error, and the significance of differences between the analyzed groups were evaluated using one-way analysis of variance and the Mann–Whitney U test. The data from the multiplex cytokine bead array assay were log-transformed and analyzed via multiple *t* test with false discovery rate correction consisting of the two-stage linear step-up procedure of Benjamini, Krieger and Yekutieli (*Q*: 1%). All analyses were done with GraphPad Prism 7 (GraphPad Software, Inc.), version 7.04. Differences with *p* < 0.05 were considered significant.
